# Predictors of Early Language Outcomes in Children with Connexin 26 Hearing Loss across Three Countries

**DOI:** 10.3390/children9070990

**Published:** 2022-07-01

**Authors:** Daniel Holzinger, Magdalena Dall, Sandra Kiblböck, Evelien Dirks, Peter Carew, Libby Smith, Lilian Downie, Daisy A. Shepherd, Valerie Sung

**Affiliations:** 1 Institute of Neurology of Senses and Language, Hospital of St. John of God, 4020 Linz, Austria; daniel.holzinger@bblinz.at (D.H.); sandra.kiblboeck@bblinz.at (S.K.); 2 Research Institute for Developmental Medicine, Johannes Kepler University, 4020 Linz, Austria; 3 Institute of Linguistics, University of Graz, 8010 Graz, Austria; 4 Dutch Foundation for the Deaf and Hard of Hearing Child, 1073 GX Amsterdam, The Netherlands; edirks@nsdsk.nl; 5 Department of Psychology, Utrecht University, 3584 CH Utrecht, The Netherlands; 6 Murdoch Children’s Research Institute, Parkville, Melbourne, VIC 3052, Australia; pcarew@unimelb.edu.au (P.C.); libby.smith@mcri.edu.au (L.S.); lilian.downie@mcri.edu.au (L.D.); daisy.shepherd@mcri.edu.au (D.A.S.); valerie.sung@rch.org.au (V.S.); 7 Department of Audiology and Speech Pathology, University of Melbourne, Melbourne, VIC 3053, Australia; 8 Department of Paediatrics, University of Melbourne, Melbourne, VIC 3052, Australia; 9 Centre for Community Child Health, The Royal Children’s Hospital, Melbourne, VIC 3052, Australia

**Keywords:** hearing loss, connexin 26, GJB2, expressive vocabulary, clinical characteristics, phenotype

## Abstract

GJB2-associated hearing loss (GJB2-HL) is the most common genetic cause of hearing loss in children. However, little is known about the clinical characteristics and early language outcomes in population-oriented samples including children with different degrees of hearing loss. Insight into these characteristics are relevant for the counselling of parents. Our sample consisted of 66 children at approximately 2 years of age (17–32 months) with bilateral hearing loss due to GJB2 from three population-based cohorts in Austria, Australia and the Netherlands. Predictors of early vocabulary, including demographic, audiological, genetic and intervention variables and the role of medical comorbidities and nonverbal cognition were examined. The vocabulary scores of children with GJB2-HL were approximately 0.7 standard deviations (SDs) below the norms of children with typical hearing. Age at access to family-centered early intervention and first-born position among siblings predicted language outcomes, whereas the degree of hearing loss and genetic subtype were not significantly correlated with expressive vocabulary. In children with GJB2-HL, early access to family-centered early intervention significantly affected language outcomes at the age of two.

## 1. Introduction

One to two in every 1000 babies in high-income countries [1,2,3] are born with significant bilateral hearing loss (HL). It is estimated that up to 80% of congenital HL cases have a genetic cause [4], and approximately two thirds of these children have nonsyndromic HL (without obvious physical comorbidities). Approximately 170 loci and 120 genes have been identified thus far [5], making nonsyndromic HL a highly heterogeneous condition. Studies in Europe, North America and Asia have identified a disruption of GJB2, the gene encoding connexin 26 and the major component of gap junctions in the cochlea, as the most frequent etiology of HL [5,6].

GJB2-associated hearing loss (GJB2-HL) was first described in 1997 [7]. To date, 337 variants in GJB2 have been identified, with more than half of them having strong evidence towards pathogenicity [4,8,9]. The GJB2 variant most commonly reported among populations in Europe, Australia and North and South America is c.35delG [10]. Different distributions of genotypes have been identified, particularly in ethnic populations, with the degree and progression of HL having some correlation with the gene variant.

Understanding the impact of the genetic etiology of HL on the phenotype (e.g., the degree and progression of HL, speech perception and language development, cognitive development and medical comorbidities) is of high relevance for counselling parents who need to make informed decisions on hearing technology (hearing aid/cochlear implant) and intervention planning (e.g., type and intensity of language and communication enhancement) and who want to gain a clearer perspective of their child’s future, particularly on what to expect for their child’s future communication.

Many studies have reported on the clinical characteristics associated with GJB2-HL. However, most of them report only outcomes in cochlear-implanted children, mostly auditory (e.g., speech perception), and the samples are usually small and not population-based. As a consequence, the information about the relationship between the geno- and phenotype is still fragmentary and not sufficiently generalizable to guide management and parent counselling.

Previous literature demonstrates that language development in children with GJB2-HL is better than, or at least at the same standard, as those with congenital HL of unknown etiology [11,12,13,14,15]. A recent systematic review suggested that children with GJB2 mutation do not have significantly better prognoses in auditory performance, including speech recognition, when compared with children with either nonsyndromic HL of unknown origin or other genetic causes of HL in the absence of other neurological deficits [16]. A study on the influence of the GJB2 status on reading and nonverbal cognitive development [17], again restricted to cochlear-implanted children, showed better outcomes in children with GJB2-HL compared to those with nonsyndromic HL with other etiologies. This finding suggests the preservation of central cognitive functioning with an isolated insult of the cochlea created by GJB2 variants. In an Australian study including children with hearing aids and cochlear implants with and without connexin 26 mutations [18], no significant differences between language and speech perception outcomes were found when taking into account other factors known to affect language development (degree of hearing loss, age at fitting with hearing aids and age at cochlear implantation). Similarly, a study comparing language development in GJB2- and non-GJB2- HL in children with hearing aids, demonstrated no significant differences [19].

Despite enormous advances in the early identification and management of HL and earlier access to high-quality family-centered intervention, children with HL without additional disabilities and neurocognitive impairments on average still perform significantly below their peers with typical hearing on language measures [20,21]. In a recent North American multicenter cross-sectional study with bilateral HL varying from mild to profound, Yoshinaga-Itano et al. found a mean expressive language quotient of 77.6 (approximately 1.5 standard deviations below the norm) at the age of approximately 2 years for the 367 children with no additional disabilities [22]. Vocabulary quotients were calculated by dividing the child’s expressive vocabulary age by his/her chronological age and multiplying by 100. Factors that have been shown to affect language outcomes in children with HL are audiological (the degree of HL unaided and aided), cognitive (nonverbal intelligence and executive functioning), intervention- and family-related (age at fitting with hearing aids, age at cochlear implantation, age at enrollment in early intervention and quality and quantity of family–child interactions) [23]. Even though GJB2 mutations are the most common genetic cause of HL in children, little is known about the clinical characteristics and language outcomes in population-oriented populations including children with different degrees of HL (users of hearing aids and cochlear implants) and impacts of specific genotypes on language outcomes.

The purpose of this study (aim one) is to describe the clinical characteristics, including auditory profiles, medical comorbidities and expressive vocabulary outcomes, in a sample of approximately 2-year-old children with bilateral GJB2-HL collected from three cohorts in Europe (Austria and the Netherlands) and Australia. All three countries have very similar well-established newborn hearing screening programs, medical-audiological management and family-centered early intervention (FCEI) programs. Our second and exploratory aim (aim two) is to describe the associations of the GJB2 subtype, child, family and intervention factors with early vocabulary outcomes in the child’s dominant spoken language. Expressive vocabulary was selected as the dependent variable, because it is a primary marker of early language development, where reliable measurements by parents’ reports and direct testing were available. Furthermore, early vocabulary has been shown to be highly predictive of later vocabulary development in children with HL [24], and early language development has been found to be correlated with other language-related achievements such as reading comprehension [25]. The multicenter approach was selected to increase the total sample size of children with GJB2-HL and available data on early vocabulary outcomes. Due to the limited size of the subsamples, a statistical comparison of clinical characteristics between the three subsamples, let alone a comparison of the prediction of language, was not intended.

We hypothesize that children with GJB2-HL have all degrees of HL, few medical comorbidities and expressive vocabulary scores below those expected for children with typical hearing (hypothesis one). We further hypothesize children’s early vocabulary to be associated with the level of parental education, degree of HL, the specific genotype (35delG homozygous mutation vs. other connexin mutations), the number of medical comorbidities, nonverbal cognition, child age at access to hearing aids/cochlear implants and child age at enrollment in family-centered early intervention (hypothesis two). 

## 2. Materials and Methods

### 2.1. Participants

Child data were extracted from three population-based databanks of children with HL in Australia, Austria and the Netherlands. Children with bilateral GJB2-HL born between 2003 and 2019 (Australia and Austria) and between 2014 and 2017 (the Netherlands) who had measured outcomes on expressive vocabulary at around the age of two years (17–32 months) were included.

Child data from Australia were from the Victorian Childhood Hearing Impairment Longitudinal Databank (VicCHILD), a statewide population-based longitudinal databank open to every child with any degree and type of permanent HL residing in Victoria, Australia (population 6.7 million, approximately 80,000 births per year) [26]. Since 2012, most children were identified and approached prospectively via the Victorian Infant Hearing Screening Program, which offers hearing screening to 99.5% of Victorian newborns; some were recruited through the Caring for Hearing Impaired Children (CHIC) Clinic at Melbourne’s Royal Children’s Hospital since 2016, when the clinic was established. Recruitment through VicCHILD is ongoing and uptake rate is approximately 53%; in general, participants live in geographic areas of a higher socioeconomic status than nonparticipants. Outcomes data were collected at four key developmental stages, including at approximately 2 years old. Genetic testing for GJB2 has been routinely offered to children with bilateral sensorineural HL of all degrees, without cost to families, since 2016, through the CHIC clinic as per national guideline recommendations [27]. Before that, families could access GJB2 testing sporadically. Children with a nondiagnostic GJB2 result can access genomic sequencing to investigate other genetic causes; however, this is an out-of-pocket cost for families and, therefore, the uptake is low. Testing for common variants within the *GJB2/6* genes is performed through the sequencing of exon 2 of the *GJB2* gene and the detection of two common large deletions on the *GJB6* gene with capillary electrophoresis. Language outcomes data at age 2 years were available for 36 (44.2%) children, because prior to 2016, VicCHILD did not collect language outcomes data at that age. All children with HL who are Australian citizens can freely access government-subsidized hearing amplification, cochlear implantation and early intervention services with oral and/or sign language.

The Austrian data relate to the state of Upper Austria, with a total population of 1.5 million and approximately 15,000 births per year. The Hospital of St. John of God in Linz offers multidisciplinary diagnostic services and the only early intervention program specialized for children with HL in the state. The early intervention program follows international best practice guidelines for family-centered early intervention [28]. Due to a newborn hearing screening program with a high coverage rate (97.6%) connected to a well-established tracking system of screening ‘fails’, the annual number of children enrolled in the early intervention program is highly comparable to international epidemiological data [29]. As in Australia, data of all children born between 2003 and 2019 with GJB2-associated HL were extracted from the hospital’s databank. In Austria, genetic testing for GJB2 has been offered routinely to the whole sample of children since 2003. PCR-based sequencing, including all coding exons and accompanied intronic regions, was performed before 2019, with specific genetic analysis of GJB2 as a first step, and in the case of a nondiagnostic result, a clinical exome sequencing hearing panel could be accessed. After 2019, next-generation sequencing was used. As described above, all mutations in exons and accompanied intronic regions were analyzed before 2019. Thereafter, all exons were tested via next-generation sequencing. Over most of the time-span of data collection, genetic examination for GJB2 was not recommended for children with mild HL. Therefore, children with mild HL were assumed to be under-represented in the Austrian subsample of this study. Genetic testing is free of charge in the Austrian health system. Two children had to be excluded due to a lack of language data at the age of two years. 

The Dutch data relate to the province of North Holland of the Netherlands, with a total number of approximately 30,000 births per year. The Dutch Foundation of the Deaf and Hard of Hearing Child (NSDSK) is the primary provider of family-centered early intervention for children with HL and their families in this province. Due to a newborn hearing screening program with a high coverage rate (99.7%) and a high percentage of children enrolled in early intervention after detection in this province, the total cohort was assumed to be representative of all children with HL [30]. All children enrolled in the intervention program of the NSDSK born between 2014 and 2017 (*n* = 95) of whom parents reported that their child had GJB2-HL were included in this study (*n* = 6). All children enrolled in the intervention program of the NSDSK were carefully and periodically monitored for their development during intervention. In the Netherlands, family-centered early intervention is only provided to children with bilateral and moderate to profound HL. Therefore, children with unilateral and mild degrees of HL were assumed to be under-represented in the Dutch subsample of this study. Genetic testing for GJB2 has been routinely offered to children with moderate to profound bilateral sensorineural HL without cost to families, since 2012, through the national guideline recommendations [31]. Genetic sequencing of the coding regions and accompanying intronic regions of the GJB2 and GJB6 gene is initially performed. When no causative variant is identified, the next step is exome sequencing focusing on more than 250 deafness genes that are associated with nonsyndromic as well as syndromic hearing loss; this is free of charge. One child with GJB2-associated HL had to be excluded due to a lack of expressive language data at the age of 2 years.

### 2.2. Measures

#### 2.2.1. Demographic Measures

Demographic measures were directly obtained from parents for the Australian sample, extracted from medical and intervention reports for the Austrian sample and from intervention reports for the sample from the Netherlands. Demographic information included information on the child (sex, age at diagnosis of HL) and the family (1 or 2 caregivers/parents, number of children, birth order of child with HL, family history of HL, parental education, parents’ place of birth, primary family language and use of sign language with the child with HL).

#### 2.2.2. Hearing Loss Characteristics 

The degree of HL (mild, moderate, severe, profound) at age of diagnosis was reported either from frequency-specific ABR (auditory brainstem response) or ASSR (auditory steady-state response) in Australia, Austria and the Netherlands, or by parent report in Australia. As exact hearing thresholds at the age of diagnosis were not available for the majority of the sample, the degree of HL was reported as categorical data at baseline. The pure-tone hearing thresholds for each participants around age two were determined from behavioral audiometry closest to the age of two years for all nonimplanted ears. For implanted ears, behavioral measures before implantation (if available) or results of auditory brainstem response (ABR) were used. In cases where there was a lack of auditory brainstem responses > 95 dB, a default value of 100 dB was entered. Hearing thresholds (aided or unaided) at approximately 2 years of age were reported as averages at 0.5, 1, 2 and 4 kHz (four-frequency average hearing loss (4FHL)) in the better ear. Following the WHO categorization, severity of HL was determined by the average pure-tone thresholds as follows: 26–40 dB was defined as “mild”, 41–60 dB as “moderate”, 61–80 dB as “severe” and ≥81 dB as “profound”. HL was defined as symmetrical if the average pure-tone threshold of low (0.5 and 1 kHz) frequencies was within 20 decibels of the high (2 and 4 kHz) frequencies on the same ear. Progressive HL was determined by change in categories of HL (mild, moderate, severe, profound) between the two audiology measurements (at the time of diagnosis and the time of language assessment at approximately 2 years). Change to a higher degree of loss was defined as “progressive”, change to a lesser degree of loss was defined as “improved” and no change in category was described as “unchanged”. Information about the use of hearing aids and/or cochlear implants was included.

#### 2.2.3. Genetic analyses

GJB2 mutation subgroups were defined in three groups: 35delG homozygous, 35delG compound heterozygous and “other”, following Cryns et al.’s (2004) classification of GJB2 subgroups [32]. The national procedures of genetic analyses offered to children with HL were described in the participant section above.

#### 2.2.4. Medical and Developmental Measures

Perinatal information (gestational age, birth weight, neonatal intensive care unit (NICU) admission, resuscitation) was reported by parents (Australia, the Netherlands) or directly extracted from available medical records (Austria). Australian parents reported additional medical diagnoses according to a list of 16 medical comorbidities (seasonal allergies, asthma or other lung problems, heart problems, kidney problems, immune disorders, diabetes or high blood sugar, cancer, arthritis, frequent of very bad headaches, epilepsy or seizures, serious stomach or bowel problems, emotional or mental health problems such as depression or anxiety, problems with alcohol or drugs, intellectual disabilities, autism spectrum disorders, learning disorders, attention disorders (ADHD or ADD) and a free-entry field). The same additional medical diagnoses categories were used for data extraction from patient charts in Austria and the Netherlands.

For the assessment of nonverbal cognitive development, the cognition scale of Bayley III (German version of the Bayley Scales of Infant and Toddler Development, Third Edition) [33] was used in Austria. For the Dutch sample, the Bayley cognition scores were available for two children and the Snijders-Oomen nonverbal intelligence scale (SON-R) [34] was used to assess nonverbal cognition in the other three children. For the Australian sample, no data of direct cognitive assessment around the age of two years were available.

#### 2.2.5. Age at Access to Intervention

Intervention included provision with hearing aids and/or cochlear implants, as well as early intervention with the aim to increase parental self-efficacy: supporting families with the use of hearing technology, their coping with the diagnosis and the adaptation of the quality and quantity of communication and language to the needs of their child. As all three countries implement modern family-centered approaches of early intervention, the current study did not include specific details on early intervention programs, but reported on the child’s age at provision with hearing aids/cochlear implants and age at entry to family-centered early intervention.

#### 2.2.6. Language Measures

In Australia, expressive vocabulary was measured with the Sure Start Language Measure (SSLM) [35], a validated 100-word caregiver checklist based on the MacArthur Bates Communicative Development Inventory, UK Short Form (MCDI:UKSF) [36], with high reliability and concurrent validity. The mean standard score was 100 with a standard deviation of 15.

In Austria, two measures were used to assess expressive vocabulary. The subtest “word production” of the SETK-2 [37] is a standardized direct measurement of a child’s skill of naming objects and pictures. The subtest “word production” of the SETK-2 shows good reliability with a Cronbach’s Alpha of 0.88. The toddler form of the MCDI [38] is a standardized parent questionnaire with high reliability and validity that assesses the expressive vocabulary by use of an extensive word list. We used a translation into Austrian German by Marschik et al., Austrian Communicative Development Inventory (ACDI) [39]. In clinical practice, the MCDI parent word list was particularly used when expressive vocabulary skills were estimated to be too low to gain differentiated results by use of the SETK-2. A study comparing results of direct measurements and parent reports demonstrated high correlations (r = 0.87) between SETK-2 (subtest word production) and a German version of the MCDI (ELFRA-2) [40].

In the Netherlands, expressive language was measured with the “Schlichting Expressive Language Test” (SELT) [41]. The subtest for expressive vocabulary was used, where children had to name pictures shown to them. The test consisted of 70 items that had to be named correctly for a positive score (range total score was 0–70). The test ended when five successive items were wrong. Raw scores were transformed into standardized Q-scores with a mean score of 100 (SD = 15; normal range 85–115), based on norm data per age group. Internal consistency of the test was 0.89 (lambda-2).

We presented the expressive vocabulary standard scores and percentiles provided by the language measures. To make the different measures comparable, standardized test scores were separately converted into z-scores using the formula; z = (x − μ)/σ, where x is the raw score, μ is the population mean and σ is the population standard deviation. The Sure Start Language Measure and the Schlichting expressive language score were converted from standard scores (μ = 100, σ = 15). SETK-2 was converted from T-scores (μ = 50, σ = 10) and the ACDI score was converted from percentile scores.

### 2.3. Statistical Analysis

To address aim 1, descriptive statistics were used to summarize demographic, hearing, genetics, medical and developmental characteristics, intervention characteristics and language outcomes across the three cohorts separately and combined. Means and standard deviations were reported for normally distributed continuous data, medians and ranges for skewed continuous data and counts and proportions for categorical data.

For aim 2, simple linear regression was used to describe the relationship between language score and individual predictors identified a priori. The predictors included demographic characteristics (sex, age at assessment, age at diagnosis of HL, the child’s birth order among siblings, family history of HL, maternal education, multilingual background, use of signed language), characteristics of HL (4 levels from mild to profound at diagnosis and 4FHL at assessment, change between categories of HL from first diagnosis to age at language assessment), medical (additional medical diagnoses) and developmental (nonverbal cognition), as well as intervention characteristics (age at first hearing aid fitting, fitting of the speech processor of a cochlear implant, age at enrollment into an early intervention program) and genetic subgroup. Regression analyses were performed for the total sample, with the R-squared and F-statistic reported to summarize the predictive ability. In addition, regression coefficients for each univariate predictor (alongside their 95% confidence intervals) were reported to describe the associations between each predictor variable and language score.

## 3. Results

Sixty-six participants with GJB2 mutations from three geographic regions (Australia *n* = 34; Austria *n* = 27; the Netherlands *n* = 5) were described. 

### 3.1. Participant and Family Demographic Characteristics

Most children were male, and almost a fifth of the sample had a family history of HL (HL in parents 15.6%, HL in siblings 12.2%; Table 1). There was an over-representation of highly educated parents in the Australian cohort. The majority of children were either born in Europe or Australia/New Zealand. The proportion of children whose first spoken language was a minority language was 15%, with a higher proportion in Australia as compared to Austria. Signed communication was used by approximately a quarter of the total sample, almost exclusively in the form of sign-supported speech, where spoken language and a signed variant of that language were used simultaneously. 

### 3.2. Hearing Loss Characteristics

The degree of HL at diagnosis ranged from mild to profound, with the majority of children having a severe (20.3%) or profound (43.8%) loss (Table 2). In Austria, connexin testing is not recommended to parents of children with mild HL, and in the Netherlands, children with mild HL are not enrolled in FCEI; therefore, this group may be under-represented. The higher severity in the degree of HL was evident due to the high median 4FLH score of 74.1 dB. Approximately half of the sample used a cochlear implant (either bi- or unilaterally), just over half used hearing aids and 9.1% used a hearing aid and a cochlear implant concurrently.

The hearing severity for the majority of the sample (75%) did not change between the diagnosis and language assessment at age 2 years (Table 2). Eleven participants showed evidence of a progressive loss at the categorical degree of loss level (17.2% of the total sample). Five participants showed an improvement in their hearing levels.

Of the 42 participants with sufficient audiometric data, eight (19.0%) had high-frequency average (2 kHz and 4 kHz) hearing levels at least 20 dB worse than low-frequency average (0.5 kHz and 1 kHz) hearing levels. Four children had this high-frequency sloping loss bilaterally, three showed this pattern in the right ear only and one had this pattern in the left ear only. All data on the audiometric shape were derived from behavioral assessments.

Six participants (14.2%) demonstrated an asymmetry between their ears at the categorical degree of HL level. Five out of thirty participants with behavioral assessment data demonstrated this asymmetry. In each instance, the left ear was poorer than the right ear (two participants left ear moderate, right ear mild; two participants left ear severe, right ear moderate; one participant left ear profound, right ear moderate). One of the twelve participants with electrophysiological data demonstrated an asymmetry, again with the left ear being poorer (moderate) than the right ear (mild).

### 3.3. Genetic Characteristics

Genetic reports detailing connexin subtypes were available for approximately 70% of the children (Table 3). For the other 20 children, parents reported a connexin mutation to be the cause of their child’s hearing loss. Whereas, in the Australian sample, a high rate (41.4%) of the 35delG compound heterozygous variant was found, the 35delG homozygous variant was the most frequent (70.6%) in Austria.

Clinical characteristics of children with the three categories of GJB2 mutation are listed in Table 4. The 35delG homozygous group had higher proportions of children with more severe degrees of HL and cochlear implant users. In all three categories, most children had stable HL and did not have any additional medical diagnoses.

### 3.4. Medical and Developmental Characteristics

Three children (5%) were admitted to neonatal special or intensive care, and two children (3%) required resuscitation. Fifteen percent of the total sample had one additional medical diagnosis in addition to HL. Two children (3%) had two or more additional medical diagnoses. Developmental data around the age of two years derived from direct testing were available for most children of the Austrian and Dutch cohorts (74.1% and 100%, respectively). The average score for nonverbal cognitive functioning was almost half a standard deviation above average.

#### Intervention Characteristics

Diagnosis of HL was very early across all cohorts (Table 5), with a median age of 1 month for the cohorts combined. Children tended to be enrolled in early intervention young (median age 4 months, combined cohort), with an early provision of hearing aids and/or cochlear implants (Table 6). 

### 3.5. Expressive Vocabulary Outcomes

Expressive vocabulary average standard scores measured with the Sure Start Language Measure in the Australian sample and with the Schlichting expressive vocabulary test in the Dutch sample were substantially below the norms (approximately 1 SD) (Table 6). For the Austrian sample, standardized direct testing was only used for linguistically more advanced children, whereas for half of the sample, parents completed the MCDI questionnaire. The mean expressive vocabulary scores were below their respective norms for each cohort separately (Table 7). For the combined sample, the language Z-scores were, on average, 0.7 standard deviations below the norm, indicating lower performance than expected for same-aged peers without hearing loss. Low language scores (<1 SD below the mean) were observed in 40% of the total sample, with similar proportions across each cohort separately. 

## 4. Associations between Expressive Language Scores and Demographic, Clinical and Intervention Characteristics

### 4.1. Demographic Characteristics and Language Outcomes

Neither sex (*F*(1, 64) = 0.04; *R*^2^ =0.000; *p* = 0.840) nor age (*F*(1, 64) = 1.09; *R*^2^ = 0.017; *p* = 0.301) were predictive of expressive language score at 2 years of age, with females tending to have slightly lower scores than males (coefficient= −0.06; 95% CI (−0.70, 0.57)) and language z-scores tending to increase with increased age at assessment (coefficient = 0.05; 95% CI (−0.05, 0.14)). Neither maternal education (*F*(2, 58) = 1.25; *R*^2^ = 0.041; *p* = 0.700), nor family history of HL (*F*(1, 62) = 0.08; *R*^2^ = 0.001; *p* = 0.774) were predictive of expressive language scores at 2 years of age, with language z-scores tending to increase with higher levels of maternal education (coefficient= 0.21; 95% CI (−0.86, 1.27)) and language z-score tending to be lower if a family history of HL was present (coefficient = −0.21; 95% CI (−0.82, 0.61)).

Neither a bilingual home environment (*F*(1, 49) = 1.22; *R*^2^ = 0.024; *p* = 0.275) nor the use of signed language at home (*F*(1, 64) = 0.19; *R*^2^ = 0.003; *p* = 0.668) were predictive of an expressive language score at 2 years of age, with language z-scores tending to be higher in families that used only one language at home (coefficient = −0.41; 95% CI (−1.15, 0.33)) and lower in those using sign language at home (coefficient = 0.17; 95% CI (−0.62, 0.96)). The order the child was born in the family predicted the expressive language scores at age 2 years (*F*(3, 60) = 2.59; *p* = 0.036, with an *R*^2^ of 0.110), with the language z-score decreasing by 0.70 (95% CI (−1.35, −0.05)) for each increase in birth order number.

### 4.2. Hearing Loss Characteristic and Language Outcomes

The degree of HL at diagnosis (four levels from mild to profound) was not predictive of the language score at age 2 years, (*F*(3, 62) = 1.37; *R*^2^ = 0.062; *p* = 0.234), nor was the degree of HL at assessment (*F*(3, 60) = 0.46; *R^2^* = 0.023; *p* = 0.301) or 4FHL at assessment (dB HL), (*F*(1, 61) = 0.18; *R*^2^ = 0.003; *p* = 0.669). The language z-score tended to be higher with a lesser degree of hearing loss at diagnosis (coefficient = 0.60; 95% CI (−0.39, 1.59)), lesser degree of hearing loss at assessment (coefficient = 0.57; 95% CI (−0.52, 1.67)) and the same within 4FHL (coefficient = 0.00; 95% CI (−0.01, 0.01)). Devices worn (no device, hearing aids or cochlear implants) was not predictive of expressive language at age 2 years (*F*(3, 63) = 0.30; *R*^2^ = 0.010; *p* = 0.482), with the language z-score tending to be higher in children with no devices (coefficient = 0.44; 95% CI (−0.80, 1.67)).

### 4.3. GJB2 Subgroups and Language Outcomes

The association between expressive vocabulary and the connexin mutation subgroup was explored using a regression analysis. The connexin mutation subgroup was not predictive of the expressive language score (*F*(2, 43) = 0.26; *R*^2^ = 0.012; *p* = 0.668), with the language z-score tending to be lower in the 35DelG homozygous subgroup (coefficient = 0.17; 95% CI (−0.68, 1.03)). 

### 4.4. Medical Characteristics, Nonverbal Cognition and Language Outcomes

**Figure 1:**
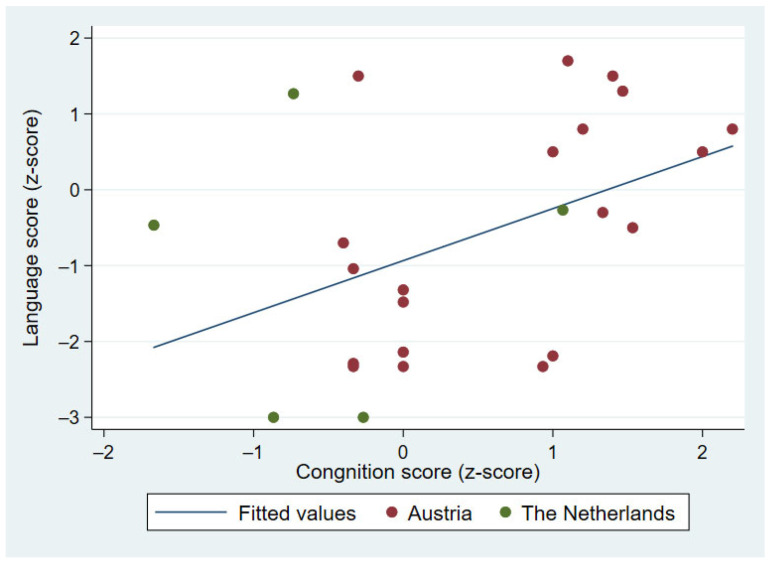
Association between expressive vocabulary and nonverbal cognition z-scores.

The number of additional medical diagnoses was available for Australian and Austrian participants. Number of medical diagnoses was not predictive of expressive language at age 2 years (*F*(1, 57) = 1.58; *R*^2^ = 0.027 *p* = 0.213), with the language z-score tending to be lower in children with one or more additional medical diagnosis (coefficient = 0.40; 95% CI (−1.04, 0.24)). The cognition score, available for participants from Austria and the Netherlands, predicted an expressive language score in the Austrian group (*F*(1, 18) = 7.51; *p* = 0.013), with *R*^2^ of 0.290 and also when the groups were taken together (*F*(1, 23) = 5.51, *p* = 0.028), with *R*^2^ of 0.190. The language z-score increased by 0.68 (95% CI (0.08, 1.29)) for each one-point increase in the cognition z-score. The correlation is illustrated in Figure 1.

### 4.5. Intervention Characteristics and Language Outcomes

The age of hearing aid fitting was found to be weakly predictive of the expressive vocabulary score in the Austrian group (*F*(1, 23) = 3.33; *R*^2^ = 0.126; *p* = 0.081) and in the group as a whole (*F*(1, 57) = 2.99; *R*^2^ = 0.050; *p* = 0.089) without reaching a significance level. The language z-score tended to be higher with lower age of hearing aid fitting (coefficient = −0.05; 95% CI (−0.11, 0.01)). Age at first cochlear implant was not predictive of the expressive language score (*F*(1, 30) = 0.56; *R*^2^ = 0.018; *p* = 0.459), with the language z-score tending to be higher with lower age of cochlear implantation (coefficient = −0.02; 95% CI (−0.09, 0.04)).

**Figure 2:**
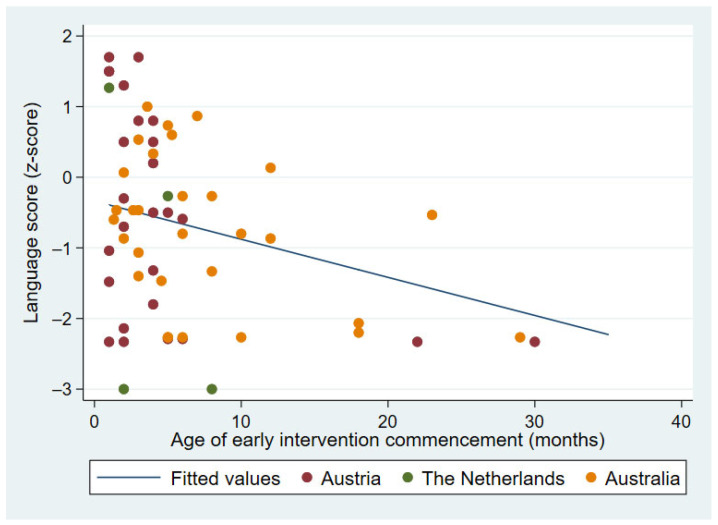
Association between expressive vocabulary and age at enrollment in early intervention.

Of note, the age at commencement of family-centered intervention was found to predict expressive language (*F*(1, 58) = 9.33; *p* = 0.003) with *R*^2^ of 0.140. The language z-score increased by 0.07 (95% CI (−0.10, −0.01)) for each 1-month decrease in age of commencing early intervention. The correlation is illustrated in Figure 2. 

## 5. Discussion

This study described the clinical characteristics and early expressive vocabulary outcomes of children with GJB2-HL drawn from three population-oriented cohorts. In addition, a variety of predictors of early vocabulary at age of approximately 2 years, including demographic, audiological, genetic and intervention variables and the role of medical comorbidities and nonverbal cognition were examined in exploratory analyses, given the small sample size. 

In this sample of children, the GJB2 mutation affected children with all degrees of HL, from mild to profound (hypothesis one), with a high proportion of children with profound HL (approximately 40%). The low percentage of children with mild HL (approximately 15%) may be associated with the tendency to recommend genetic testing for more severe degrees of HL in Austria in the past, and the exclusion of mild degrees of HL from early intervention programs in the Netherlands. However, previous studies have similarly reported higher percentages of children with severe and profound HL than mild HL in patients with GJB2-HL [42]. Our findings confirmed the predominance of the 35delG subtype in Europe and the typical association with profound HL [10,43,44]. In our sample, 15% of children experienced HL progression to a more severe degree. The definition of progression was based on a change in the category of HL (four levels) by parent reports where detailed audiological data were not available. In addition, changes in the hearing threshold might have been due to an imprecision and variance of diagnostic results at this early stage of life. Therefore, our findings should be interpreted with caution, even though the rates of progressive HL resembled those of larger studies. A meta-analysis of 28 studies reported HL progression in 18.7% of a sample of 1140 children [10]. The rate of asymmetrical HL in our total sample (14.2%) was based on a restricted sample of children with available behavioral assessment data. Our findings confirmed the findings of a meta-analysis of 19 studies, encompassing 801 probands with GJB2-HL and with a rate of asymmetry of 14.2% (range 0–55.6%) [10]. High-frequency HL has been reported as a characteristic feature in children with GJB2-HL [45]. In our total sample, eight children (19%) demonstrated a high-frequency sloping HL of at least 20dB in at least one ear. A comparison with previous literature would be complicated due to different definitions of high-frequency HL, imprecise definitions of cohorts and the lack of audiological data above 4 kHz in the present study.

In our sample, less than 20% of children had additional medical diagnoses; this is much less than in general populations of children with HL, where additional disabilities have been reported to be approximately 40% [46,47]. Nevertheless, even though there was no direct relationship between GJB2 disruption and nonhearing-related disorders, additional disabilities that can effect cognitive, linguistic and psychosocial development cannot be ruled out by the presence of GJB2 mutations [48,49].

Expressive vocabulary scores at the age of approximately two years were approximately 0.7 standard deviations (SD) below the norms of children with typical hearing, and approximately 40% of the total sample demonstrated scores of at least -1 SD. As compared to a recent multicenter study of 367 children with bilateral HL without additional disabilities, where the mean verbal quotient (for expressive vocabulary) at the age of approximately two years was 76.3, equivalent to almost -1.5 SDs, our data showed a less pronounced delay in vocabulary development [22]. As expected, our sample of children had lower vocabulary scores compared to children with typical hearing, thus, confirming part of hypothesis two. However, the delay was less pronounced than in populations of children with HL of different causes, even after the exclusion of those with additional disabilities.

Among the factors assumed to influence early vocabulary outcomes (second part of hypothesis two), parental education was not confirmed as a significant predictor. Similar to other studies reporting on outcomes of modern family-centered intervention programs, this finding might be due to a levelling effect of these programs supporting parents with low education to use a high-quantity and quality of parent–child interactions [50]. The language advantage of first-born children most likely reflects the children’s undivided attention and interaction time of their parents [51,52]. Additional medical diagnoses were not significantly associated with language outcomes, but the number of children with additional medical diagnoses and particularly diagnoses that impact language development was very limited in our sample. Where there was available data on standardized nonverbal cognition testing in the Austrian and Dutch samples (n = 25), nonverbal cognitive development was positively associated with expressive vocabulary scores as anticipated, and shown by other studies on predictors of language development in children with HL [47]. Contrary to expectation and unlike previous studies [22,53], the degree of HL at diagnosis and at time of assessment did not significantly predict language outcomes. This remarkable finding might be related to the advanced systems of early identification and intervention in the three countries included in the study, providing well-fitted hearing technology and parent support universally from a very early age. Similar to our findings, Cupples et al. [47] did not find a significant correlation between the degree of HL and parent-reported expressive language outcomes at the age of five years.

The most outstanding finding from our study was that the earlier the age at enrollment in early intervention, the higher the expressive vocabulary scores. A median of 4 months at enrolment in early intervention showed that the majority of children entered early intervention before the benchmark of 6 months, as recommended by international guidelines [54]. The lack of a significant correlation between age at hearing aid fitting and language development contrasted earlier findings [53,55]. In the Australian study of Ching et al., the median age of hearing aid fitting was 5.1 months, compared to our study’s median hearing aid fitting age of 3.0 months. Our small sample size and little variation within the group could have contributed to the lack of significance. Interestingly, in our sample, age at diagnosis of HL was not found to be related to language outcomes. Our findings confirmed those of a number of other studies [22,56], demonstrating significant effects of age at early intervention enrollment on language outcomes for the group of children with GJB2-HL. Even in those children with nonverbal cognitive strengths and a low number of additional medical diagnoses, timely access to family-centered early intervention likely impacts language development. Early provision with hearing technology needs to go hand in hand with the support of parents in the management and their child’s acceptance of the devices. Family-centered early intervention can help parents in coping with their child’s diagnosis and to gain a new and positive perspective for their child and family, which also leads to increased self-efficacy [57] and involvement in the intervention. Essentially, family-centered early intervention supports parents in the use of language and communication optimally adapted to their child’s communication needs. The effect of the amount and quality of parental language with a child with HL has been shown to be strongly related to language development [58,59,60]. In families with a child with HL in the second or later position among siblings, special attention to individual parent–child interactions with this child should be emphasized.

### Strengths and Limitations

This study spanned three population-based cohorts over three countries, and included children with all degrees of bilateral HL with a GJB2 mutation. Clinical characteristics described in the study included information on the child’s hearing characteristics, genetic subtype, medical diagnoses and nonverbal cognition. Early vocabulary was measured by the use of reliable standardized instruments. In addition to child clinical characteristics, the role of environmental factors related to the family and intervention for the prediction of early language development was examined.

Several limitations need to be mentioned. Although the samples of children were derived from population-based cohorts, genetic testing may have only been performed for a proportion of, rather than all, participants, with a possible bias towards more severe degrees of HL. Children with unilateral HL were not included, because genetic testing is not generally recommended for this subgroup of children. The limited sample size restricted the generalization of our results to the whole population of children with GJB2-associated HL, especially for the regression analyses of predictor–outcome associations, which require replication in larger samples that can account for between-country factors. Another possible limitation is the use of different instruments for language assessments (by parent reports in Australia, direct assessment in the Netherlands, combination of parent reports (in children with very low expressive vocabulary) and a direct assessment in Austria). However, high correlations between the different types of language measures of expressive vocabulary at age 2 have been reported in the literature [39]. Due to parent-reported audiological data at the time of diagnosis in Australia, the category of HL (from mild to profound) rather than the exact pure-tone threshold was used in the analyses. Due to the restriction of parent-reported HL categories at the time of diagnosis, the exploration of progressive HL was limited; we recorded change across categories of HL levels and could not explore change according to actual hearing thresholds. Nonverbal cognition data were only available for the Austrian and Dutch cohorts. 

## 6. Conclusions

Children with GJB2- HL across three countries demonstrated expressive vocabulary scores at the age of two approximately 0.7 SD below the norm for children with typical hearing. Among the child-related audiological and genetic subtype and medical and developmental variables, only nonverbal cognition was found to predict expressive vocabulary. In addition, being the first child in the family and early enrollment in early intervention showed significant positive effects on language development.

## Figures and Tables

**Table 1:** Participant and family demographic characteristics across the three cohorts separately and combined.

	Australia	Austria	Netherlands	All
	*n* = 34	*n* = 27	*n* = 5	*n* = 66
Child				
Sex, male—*n* (%)	20 (58.8)	15 (55.6)	3 (60.0)	38 (57.8)
Age at HL diagnosis, months—median (range)	0.8 (0.2 to 1.9)	3.0 (1.0 to 29.0)	1.0 (1.0 to 2.0)	1.0 (0.24 to 29.0)
Number of children in family—*n* (%)				
1	15 (44.1)	8 (32.0)	3 (60.0)	26 (40.6)
2	12 (35.3)	14 (56.0)	1 (20.0)	27 (42.2)
3	6 (17.7)	2 (8.0)	1 (20.0)	9 (14.1)
4 or more	1 (2.9)	1 (4.0)	0 (0.0)	2 (3.1)
Order of child in family —*n* (%)				
1st	15 (44.1)	10 (40)	3 (60.0)	28 (43.8)
2nd	14 (41.2)	12 (48)	2 (40.0)	28 (43.8)
3rd	4 (11.7)	2 (8.0)	0 (0.0)	6 (9.4)
4th or more	1 (2.9)	1 (4.0)	0 (0.0)	2 (3.1)
Family history of HL *—*n* (%)				
Parent	5 (14.7)	5 (20.0)	0 (0.0)	10 (15.6)
Sibling	3 (8.8)	5 (20.8)	0 (0.0)	8 (12.7)
Maternal education—*n* (%)				
Low	2 (6.1)	3 (12.5)	1 (25.0)	6 (10.7)
Medium	8 (24.2)	19 (79.2)	2 (50.0)	24 (42.9)
High	23 (69.7)	2 (8.3)	1 (25.0)	26 (46.4)
Primary language spoken at home—*n* (%)				
Majority language	19 (55.9)	14 (51.9)	1 (20.0)	1 (1.5)
Bilingual (two or more spoken languages)	9 (26.5)	3 (11.1)	1 (20.0)	10 (15.5)
Other language only	2 (5.9)	0 (0.0)	2 (40.0)	6 (9.1)
Sign language	0 (0.0)	2 (7.4)	0 (0.0)	2 (3.0)
Sign-assisted language	4 (11.8)	8 (29.6)	1 (20.0)	13 (19.7)

* Family history of HL of any age onset.

**Table 2:** Participants’ hearing loss characteristics.

	Australia	Austria	Netherlands	All
	*n* = 34	*n* = 27	*n* = 5	*n* = 66
Age at hearing assessment (months)—mean (SD)	22.0 (21.1)	24.6 (4.4)	30.7 (7.6)	23.5 (16.3)
Type of loss—*n* (%)				
Sensorineural	33 (97.1)	27 (100.0)	5 (100.0)	65 (98.5)
Mixed	1 (2.9)	0 (0.0)	0 (0.0)	1 (1.5)
Degree of loss at diagnosis (in the better ear)—*n* (%)				
Mild	9 (26.5)	1 (3.7)	0 (0.0)	10 (15.2)
Moderate	7 (20.6)	10 (37.0)	1 (20.0)	18 (27.3)
Severe	5 (14.7)	5 (18.5)	2 (40.0)	12 (18.2)
Profound	13 (38.2)	11 (40.7)	2 (40.0)	26 (39.4)
Degree of loss at assessment (in the better ear)—*n* (%)				
Mild	7 (21.9)	2 (7.4)	0 (0.0)	9 (14.1)
Moderate	6 (18.6)	7 (25.9)	1 (20.0)	14 (21.9)
Severe	4 (12.5)	7 (25.9)	2 (40.0)	13 (20.3)
Profound	15 (46.9)	11 (40.7)	2 (40.0)	28 (43.8)
4FHL—mean (SD)	73.5 (31.0)	74.3 (25.0)	76.9 (24.9)	74.1 (27.8)
Change in degree of loss from diagnosis to assessment (dbHL)—*n* (%)				
Stayed the same (unchanged)	23 (71.9)	20 (74.1)	5 (100.0)	48 (75.0)
Improved	2 (6.3)	3 (11.1)	0 (0.0)	5 (7.8)
Worsened (progressive)	7 (21.9)	4 (14.8)	0 (0.0)	11 (17.2)
Hearing aid				
Current use—*n* (%)	16 (47.1)	17 (63.0)	3 (60.0)	36 (54.6)
Cochlear implant				
Unilateral, *n* (%)	1 (2.9)	5 (18.5)	0 (0.0)	6 (9.1)
Bilateral, *n* (%)	15 (44.1)	8 (29.6)	2 (40.0)	25 (38.5)
Concurrent hearing aid and cochlear implant use—*n* (%)	1 (2.9)	5 (18.5)	0 (0.0)	6 (9.1)
No device	3 (8.8)	2 (7.4)	0 (0.0)	5 (7.6)

**Table 3:** Participants’ genetic characteristics.

Connexin	Australia (VicCHILD)	Austria	Netherlands	All
	*n* = 34	*n* = 27	*n* = 5	*n* = 66
Genetics report, detailing subtypes available—*n* (%)	29 (87.9)	17 (63.0)	0 (0.0)	46 (69.7)
Connexin mutation type—*n* (%)				
c.35delG homozygous	7 (24.1)	12 (70.6)		19 (41.3)
c.35delG comp. heterozygous	12 (41.4)	3 (17.7)		15 (32.6)
Other	10 (34.5)	2 (11.8)		12 (26.1)

**Table 4:** Hearing loss and other medical characteristics of participants with different subtypes of GJB2 mutation.

GJB2	35delG Homozygous	35delG Compound Heterozygous	Other
	*n* = 19	*n* = 15	*n* = 12
Degree of HL at Ax—*n* (%)			
Mild	0 (0.0)	2 (14.3)	3 (27.3)
Moderate	2 (10.5)	5 (35.7)	2 (9.1)
Severe	5 (26.3)	0 (0.0)	4 (36.4)
Profound	12 (63.2)	7 (41.67)	3 (27.3)
Uses cochlear implant/s—*n* (%)	13 (68.4)	6 (40)	3 (25)
Change in degree of loss—*n* (%)			
Unchanged	14 (73.7)	12 (85.7)	7 (63.6)
Improved	0 (0.0)	1 (7.1)	0 (0.0)
Progressive	5 (26.3)	1 (7.1)	4 (36.4)
Number of other medical conditions—mean (SD)	0.7 (0.9)	0.33 (0.6)	0.0 (0.0)
None—*n* (%)	16 (84.2)	11 (78.6)	10 (83.3)
One—*n* (%)	2 (10.5)	3 (21.4)	2 (16.7)
Two or more—*n* (%)	1 (5.3)	0 (0.0)	0 (0.0)

**Table 5:** Participants’ medical and developmental characteristics.

	Australia (VicCHILD)	Austria	Netherlands	All
	*n* = 34	*n* = 27	*n* = 5	*n* = 66
**Medical and developmental characteristics**				
Perinatal				
Birth gestation, weeks—mean (SD)	39.1 (1.6)	40.2 (1.5)	39.2 (0.8)	39.5 (1.6)
Birth weight, grams—mean (SD)	3287.5(453.5)	3525.0 (670.4)	3262.3 (650.7)	3386.6 (578.5)
Admission to neonatal special/intensive care—*n* (%)	1 (3.8)	2 (7.7)	0 (0)	3 (5.2)
Resuscitation required at birth—*n* (%)	0 (0)	2 (7.41)	0 (0)	2 (3.2)
Additional medical diagnoses—*n* (%)				
0	25 (78.1)	23 (85.2)	-	48 (81.4)
1	5 (15.6)	4 (14.8)	-	9 (15.3)
2 or more	2 (6.3)	0 (0)	-	2 (3.4)
**Cognition**				
Cognition reports available—*n* (%)	-	20 (74.1)	5 (100%)	
Cognition z-score—mean (SD)	-	0.67 (0.86)	−0.49 (1.01)	0.44 (0.99)

**Table 6:** Intervention characteristics.

	Australia	Austria	Netherlands	All
	*n* = 34	*n* = 27	*n* = 5	*n* = 66
Age at HL diagnosis, months—median (range)	0.8 (0.2 to 1.9)	3.0 (1.0 to 29.0)	1.0 (1.0 to 2.0)	1 (0.2 to 29.0)
Age of first fitting, months—median (range)	2.6 (1.0 to 24.0)	4.0 (1.0 to 30.0)	4.0 (3.0 to 24.0)	3.0 (1.0 to 30.0)
Age of first implant, months—median (range)	10.3 (6.8 to 18.0)	14.0 (12.0 to 24.0)	12.0 (12.0 to 12.0)	12.0 (6.8 to 24.0)
Age of second implant, months—median (range)	10.3 (6.8 to 20.3)	14.0 (13.0 to 28.0)	12.0 (12.0 to 12.0)	12.6 (6.8 to 28.0)
Age at start of early intervention, months—median (range)	5.1 (1.3 to 29.0)	3.0 (1.0 to 30.0)	5.0 (1.0 to 35.0)	4.0 (1.0 to 35.0)

**Table 7:** Participants’ expressive vocabulary (standard scores and percentiles) around age 2.

	Australia	Austria	Netherlands	All
	*n* = 34	*n* = 27	*n* = 5	*n* = 66
Age at language measure, months—median (range)	25.5 (23.5 to 30.9)	27.0 (17.0 to 32.0)	29.0 (24.0 to 30.0)	26.0 (17.0 to 32.0)
**Language scores**				
Sure Start Language Measure standard score—mean (SD)	89 (15.1)			
ACDI percentile—mean (SD) (*n* = 14)		7.1 (10.5)		
SETK2 t-score—mean (SD) (*n* = 13)		56.9 (8.4)		
Schlichting expressive standard score—mean (SD)			83.6 (28.0)	
**Language Z scores**				
Sure Start Language Measure standard score—mean (SD)	−0.73 (1.01)			
ACDI/SETK2– mean (SD)		−0.59 (1.46)		
Schlichting expressive standard score—mean (SD)			−1.09 (1.87)	
Total language z-score—mean (SD)				−0.70 (1.3)
**Low language scores**				
Participants with scores > 1SD below the mean—*n (%)*	12 (35.3)	12 (44.4)	2 (40.0)	26 (39.4)

## Data Availability

Data are not publicly available because we did not have consent in favor of public access of the parents of the subjects involved.
